# Crystal structure of a short-chain dehydrogenase from *Burkholderia cenocepacia* J2315 in complex with NADP^+^ and benzoic acid

**DOI:** 10.1107/S2053230X24011282

**Published:** 2024-11-30

**Authors:** Kafi K. J. Belfon, Olive Beyer, Jan Abendroth, David M. Dranow, Donald D. Lorimer, Ariel Abramov, Yazmine Latimore, Connor Hamilton, Aniah Dawkins, Isabella Hinojosa, Xavier Martinez, Sofia Mirabel, Miriam Duncan, Reagan Womack, Lillian Hicks, Zachary R. Turlington, Thomas E. Edwards, Andrew T. Torelli, Katherine A. Hicks, Jarrod B. French

**Affiliations:** a The Hormel Institute University of Minnesota Austin MN55912 USA; b Department of Chemistry and Biochemistry University of Maryland Baltimore County Baltimore MD21250 USA; c UCB BioSciences, Bainbridge Island, WA98110, USA; d Seattle Structural Genomics Center for Infectious Disease (SSGCID), Seattle, WA98105, USA; e Department of Natural Sciences Albany State University Albany GA31707 USA; f College of Health Professions, Nursing and Pharmacy Manchester University North Manchester IN46962 USA; g Department of Chemistry and Biochemistry Hampton University Hampton VA23668 USA; h Department of Integrative Biology Oklahoma State University Stillwater OK74078 USA; i Department of Biological Sciences College of the Sequoias Visalia CA93277 USA; j Department of Microbiology, Immunology and Molecular Genetics University of California Los Angeles Los Angeles CA90095 USA; k Biological and Chemical Sciences Department, Manhattan University, Bronx, NY10471, USA; l College of Natural Sciences and Mathematics Lenoir-Rhyne University Hickory NC28601 USA; m Department of Chemistry Clemson University Clemson SC29634 USA; n State University of New York at Cortland Cortland NY13045 USA; o Department of Chemistry Ithaca College Ithaca NY14850 USA; Dartmouth College, USA

**Keywords:** short-chain dehydrogenase/reductases, research experiences for undergraduates, SSGCID

## Abstract

*Burkholderia cenocepacia* is an opportunistic human pathogen that can cause lethal infections in immunocompromised individuals, particularly those with cystic fibrosis. As such, there is a critical need to identify and characterize the structure and function of enzymes that participate in the metabolic pathways of this bacterium. Here, the high-resolution X-ray crystal structure of a short-chain dehydrogenase reductase (SDR) from *B. cenocepacia* J2315 (BcSDR) in complex with the coenzyme NADP^+^ and a benzoic acid ligand is presented. This protein has the conserved Rossmann fold of the SDR superfamily and the characteristic TG*xxx*G*x*G motif of the classical SDR subfamily. However, unlike classical SDRs, the active site of BcSDR has a leucine residue in place of the highly conserved and catalytically important tyrosine residue. Sequence analysis confirms that this leucine residue is conserved in this SDR across the Burkholderiales order. This suggests that BcSDR is more appropriately classified into the divergent SDR subfamily. In addition, this enzyme would necessarily employ a different enzyme mechanism to that proposed as a general mechanism for most SDRs.

## Abstract 2

The structure of *B. cenocepacia* short-chain dehydrogenase/reductase (SDR) in complex with NADP^+^ and benzoic acid is presented. Analysis of the structure reveals a distinctive active-site architecture, suggesting that this protein is part of the divergent SDR subgroup.

## 1. Introduction

**Figure 1:**
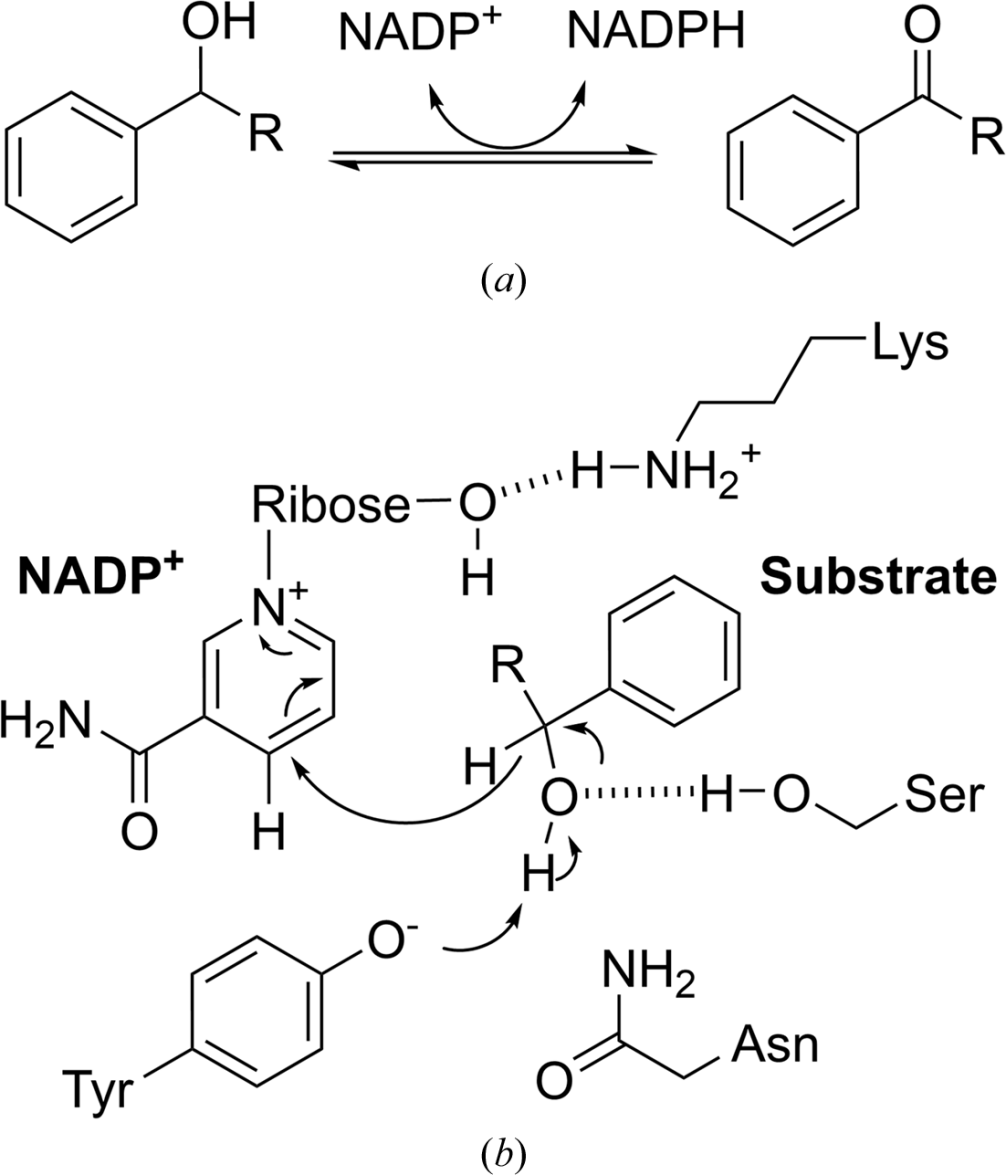
SDR chemistry. SDRs are NAD(P)-dependent oxido­reductases that catalyse a wide range of reactions, including carbonyl–alcohol oxido­reductions as shown in (*a*). Most SDRs have some variation of a Y*xxx*K active-site motif. The highly conserved tyrosine residue acts as the putative active-site base in the proposed SDR mechanism (*b*).

*Burkholderia cenocepacia* is a Gram-negative bacterium that is known to be a human pathogen. Due to its inherent antibiotic resistance, ability to form biofilms and virulence factors, this organism is often responsible for life-threatening infections in immunocompromised patients (O’Grady & Sokol, 2011 ▸; Scoffone *et al.*, 2016 ▸). Nosocomial infections caused by this organism are often spread through contaminated medical devices and between patients (Holden *et al.*, 2009 ▸; Mann *et al.*, 2010 ▸; Sass *et al.*, 2011 ▸). It is particularly harmful in cystic fibrosis patients, where it can cause respiratory infections that are difficult to treat and often fatal (Lauman & Dennis, 2021 ▸; Lipuma, 2010 ▸). The particular challenges in treating infections arising from *B. cenocepacia* arise from the natural and acquired resistance of this organism to antibiotics and the multiple resistance mechanisms that it employs (Scoffone *et al.*, 2017 ▸). To better understand molecular mechanisms of pathogenicity and to aid in the development of new treatments, the primary mission of the Seattle Structural Genomics Center for Infectious Disease (SSGCID) is to determine the 3D atomic structures of proteins and other molecules with important biological roles in human pathogens. Here, we describe the crystal structure of a *B. cenocepacia* short-chain dehydro­genase reductase (BcSDR). SDRs are a large family of NAD(P)-dependent oxidoreductases that catalyse a wide range of reactions, such as carbonyl–alcohol oxidoreductions (Fig. 1 ▸*a*). This superfamily is found in all domains of life and performs critical metabolic transformations on diverse substrates including carbohydrates, lipids, amino acids, hormones and many others (Kavanagh *et al.*, 2008 ▸). Typically, SDRs employ a conserved tyrosine residue in the dehydro­genase mechanism (Fig. 1 ▸*b*).

SDRs play important roles in varying biological processes, including hormonal signaling and regulation, detoxification of xenobiotics, lipid homeostasis and a range of metabolic functions (Kavanagh *et al.*, 2008 ▸; Mo *et al.*, 2020 ▸; Oppermann & Maser, 2000 ▸). Because of their substrate breadth and their capability to reduce C=O and C=C bonds, SDRs have also shown promise as biocatalysts (Beerens *et al.*, 2021 ▸; Borg *et al.*, 2021 ▸; Roth *et al.*, 2020 ▸). The structure described here is of an NADP-dependent SDR that is putatively involved in the metabolism of benzoic acid or its precursors. In addition to providing a basis for future drug development, the bacterial metabolism of benzoate has additional implications for human health. Because of the broad use of sodium benzoate as a food preservative, microbial benzoate catabolism by gut microbiota may influence other metabolic processes and overall human physiology (Yadav *et al.*, 2021 ▸).

## 2. Materials and methods

### 2.1. Macromolecule production

Cloning, expression and purification followed standard protocols as described previously (Bryan *et al.*, 2011 ▸; Choi *et al.*, 2011 ▸; Serbzhinskiy *et al.*, 2015 ▸). The full-length gene for the putative short-chain dehydrogenase reductase (SDR) from *B. cenocepacia* J2315 (BcSDR; UniProt B4EFS5) encoding amino acids 1–237 was PCR-amplified from genomic DNA using the primers shown in Table 1 ▸. The gene was ligation-independently cloned into pBG1861 (Alexandrov *et al.*, 2004 ▸), encoding a noncleavable N-terminal 6×His-tag. Plasmid DNA was transformed into chemically competent cells. The plasmid containing His-BcSDR was tested for expression and 2 l of culture was grown using auto-induction medium (Studier, 2005 ▸) in a LEX Bioreactor (Epiphyte Three) as described previously (Serbzhinskiy *et al.*, 2015 ▸).

His-BcSDR was purified in a two-step protocol consisting of an immobilized metal (Ni^2+^) affinity chromatography (IMAC) step followed by size-exclusion chromatography (SEC). All chromatography runs were performed on an ÄKTApurifier 10 (GE Healthcare) using automated IMAC and SEC programs (Bryan *et al.*, 2011 ▸). Thawed bacterial pellets (∼25 g) were lysed by sonication in 200 ml buffer consisting of 25 m*M* HEPES pH 7.4, 300 m*M* NaCl, 5%(*v*/*v*) glycerol, 30 m*M* imidazole, 0.5%(*w*/*v*) CHAPS, 10 m*M* MgCl_2_, 3 m*M* β-mercaptoethanol, 1.3 mg ml^−1^ protease-inhibitor cocktail (Roche, Basel, Switzerland), 0.05 mg ml^−1^ lysozyme. After sonication, the crude lysate was clarified with 20 ml (25 units µl^−1^) of Benzonase and incubated while mixing at room temperature for 45 min. The lysate was clarified by centrifugation at 10 000 rev min^−1^ for 1 h using a Sorvall centrifuge (Thermo Scientific). The clarified supernatant was then passed over an Ni–NTA His-Trap FF 5 ml column (GE Healthcare) which had been pre-equilibrated with loading buffer consisting of 25 m*M* HEPES pH 7.0, 300 m*M* NaCl, 5%(*v*/*v*) glycerol, 30 m*M* imidazole, 1 m*M* DTT. The column was washed with 20 column volumes (CV) of loading buffer and was eluted with loading buffer plus 500 m*M* imidazole in a linear gradient over 7 CV. Peak fractions were pooled and concentrated to 5 ml. A SEC column (Superdex 75, GE Healthcase) was equilibrated with running buffer consisting of 25 m*M* HEPES pH 7.0, 300 m*M* NaCl, 5%(*v*/*v*) glycerol, 1 m*M* TCEP. The peak fractions were collected and analyzed for the protein of interest using SDS–PAGE. These fractions were then pooled and concentrated to 46.9 mg ml^−1^ using an Amicon purification system (Millipore). Aliquots of 200 µl were flash-frozen in liquid nitrogen and stored at −80°C until use.

### 2.2. Crystallization

Initial crystallization trials were conducted using the sitting-drop vapor-diffusion method with the JCSG+ commercial crystallization screen (Rigaku Reagents). Each drop consisted of a mixture of 0.4 µl 23.45 mg ml^−1^ protein solution, 0.4 µl well solution and a final concentration of 5 m*M* NADP^+^. Crystals of BcSDR were obtained in screen condition C6 consisting of 40% PEG 300, 100 m*M* sodium phosphate dibasic/citric acid pH 4.2. A single crystal was directly vitrified, without additional cryoprotectant, by plunging it into liquid nitrogen prior to data collection. To facilitate phase determination by single-wavelength anomalous dispersion (SAD), a single crystal was incubated in reservoir solution supplemented with 10% 5 *M* sodium iodide in ethylene glycol, giving final concentrations of 500 m*M* sodium iodide and 10% ethylene glycol, for 30 s prior to vitrification by plunge-freezing in liquid nitrogen. Additional crystallization data can be found in Table 2 ▸.

### 2.3. Data collection and processing

X-ray diffraction data were collected at 100 K on the LS-CAT beamline 21-ID-G at the Advanced Photon Source (APS) using a Rayonix MX-300 detector. Data were integrated using *XD*S and reduced with *XSCALE* (Kabsch, 2010 ▸). Data for phasing were collected at 100 K using a Cu *K*α rotating-anode (1.5418 Å) home source and a Rigaku Saturn 944+ detector. Additional data-collection information is provided in Table 3 ▸. The raw images and detailed data-collection information are available for download at https://proteindiffraction.org/search/?q=5u4s.

### 2.4. Structure solution and refinement

The structure of BcSDR was solved by SAD phasing using data collected from crystals soaked with sodium iodide (Abendroth *et al.*, 2011 ▸). The phases were determined with *Phaser* (McCoy *et al.*, 2007 ▸) and an initial model was partially built using *ARP*/*wARP* (Perrakis *et al.*, 1999 ▸). The model was then improved through iterative rounds of model refinement using *Phenix* (Liebschner *et al.*, 2019 ▸) and manual model building with *Coot* (Emsley *et al.*, 2010 ▸). Refinement statistics are provided in Table 4 ▸. The final model was deposited in the Protein Data Bank as entry 5u4s.

## 3. Results and discussion

*B. cenocepacia* SDR (BcSDR) crystallized in an orthorhombic space group (*P*2_1_2_1_2_1_) with two molecules per asymmetric unit. The crystals had a solvent content of 41.1%, with a Matthews coefficient of 2.09 Å^3^ Da^−1^. The native data, collected on the 21-ID-G beamline at the Advanced Photon Source, were of good quality, with diffraction to 1.4 Å resolution. Because of the relatively low sequence similarity to other known structures (<32% sequence identity), experimental phasing was carried out using SAD data collected using a Cu *K*α source from crystals soaked with sodium iodide (Abendroth *et al.*, 2011 ▸). Additional data-collection and structure-solution details are provided in Tables 3 ▸ and 4 ▸.

**Figure 2:**
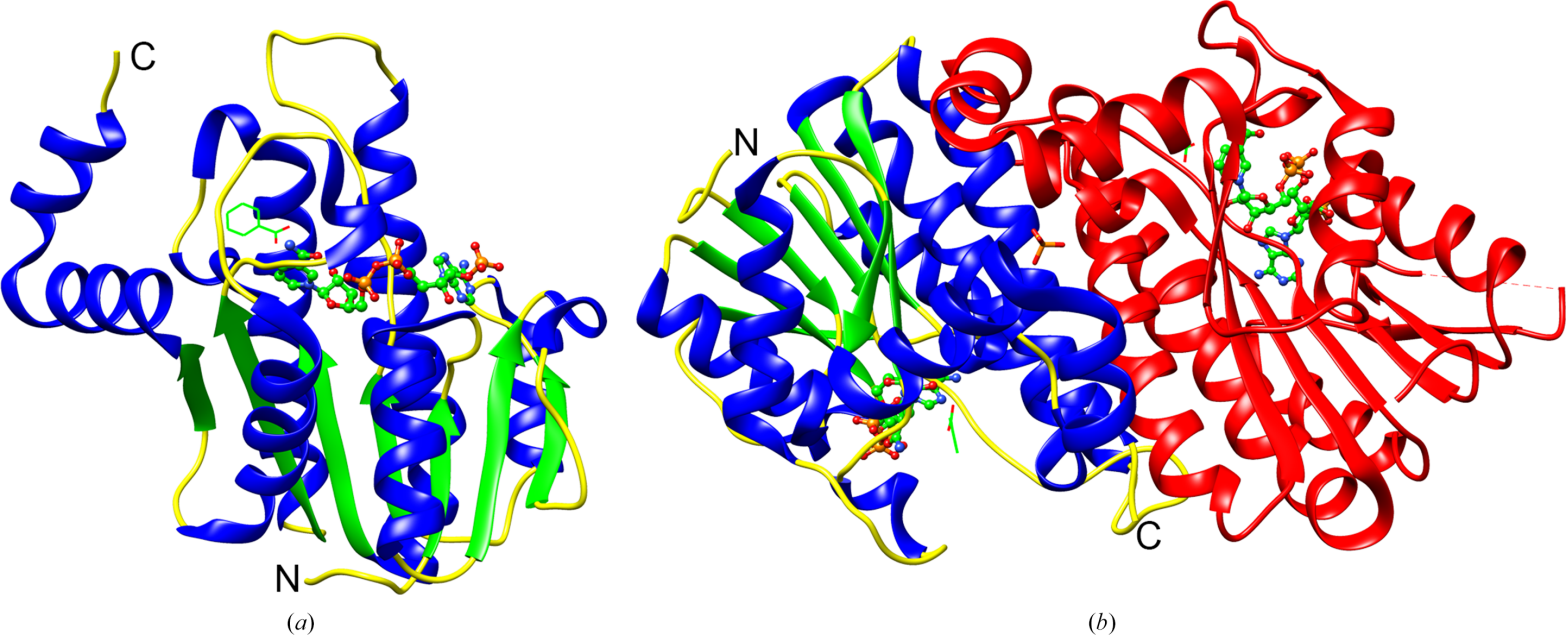
Overall structure of BcSDR. The protomer of BcSDR has a central β-sheet flanked by α-helices on each side (*a*) with NADP^+^ (ball-and-stick representation) bound in the cofactor-binding site and benzoic acid (wire representation) bound in the active site. α-Helices are shown in blue, β-sheets are shown in green and loops are shown in yellow. NADP^+^ and benzoic acid are depicted with green C atoms, red O atoms, blue N atoms and orange P atoms. The active form of BcSDR is a dimer (*b*). Chain *A*, NADP^+^ and benzoic acid are colored as in (*a*). Chain *B* is colored red.

The protomer of BcSDR has the Rossmann fold characteristic of this family of proteins, with a central parallel β-sheet flanked by α-helices on each side (Fig. 2 ▸*a*). The overall structure was observed to be homodimeric, with a phosphate ion present at the dimer interface (Fig. 2 ▸*b*). While it is possible that the phosphate ion co-purified with the protein, it is likely to be present due to the crystallization conditions, which contained 100 m*M* phosphate. Analysis using *PDBePISA* (Krissinel & Henrick, 2007 ▸) confirms the assignment of the dimer as the most stable complex, with a buried surface area between chains *A* and *B* of 11 150 Å^2^. This observation is consistent with BcSDR being part of the classical SDR subclass, the members of which are predominantly dimeric or tetrameric in structure (Kavanagh *et al.*, 2008 ▸).

**Figure 3:**
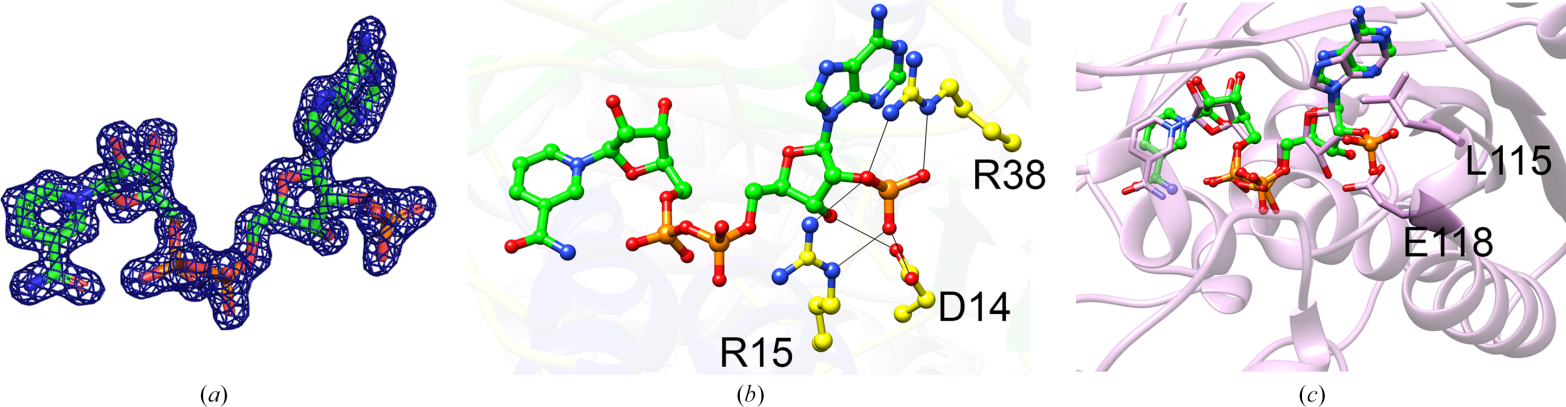
Coenzyme-binding site. The electron density (2*F*_o_ − *F*_c_, contoured at 1.5σ) around the NADP^+^ cofactor (*a*) is well resolved and clearly matches NADP^+^ (NADP in chain *A* is shown). Several key hydrogen-bonding interactions (*b*) are made between the Asp14, Arg15 and Arg38 residues of BcSDR and the phosphate moiety of NADP^+^. Here, C atoms are shown in green, O atoms in red, N atoms in blue and P atoms in orange. As demonstrated by superposition of BcSDR with an NAD-binding SDR (*c*) (PDB entry 5u8p, shown in pink with pink C atoms), residues (Leu115 and Glu118 of PDB entry 5u8p) of a nearby loop and helix provide a sterically selective force for binding NAD versus NADP. This helix that harbors Glu118 in PDB entry 5u8p is absent in BcSDR and would clash with Arg38. Note that further details about the goodness of fit of the coenzyme, including additional maps and images, can be found in the PDB validation report for PDB entry 5u4s.

**Figure 4:**
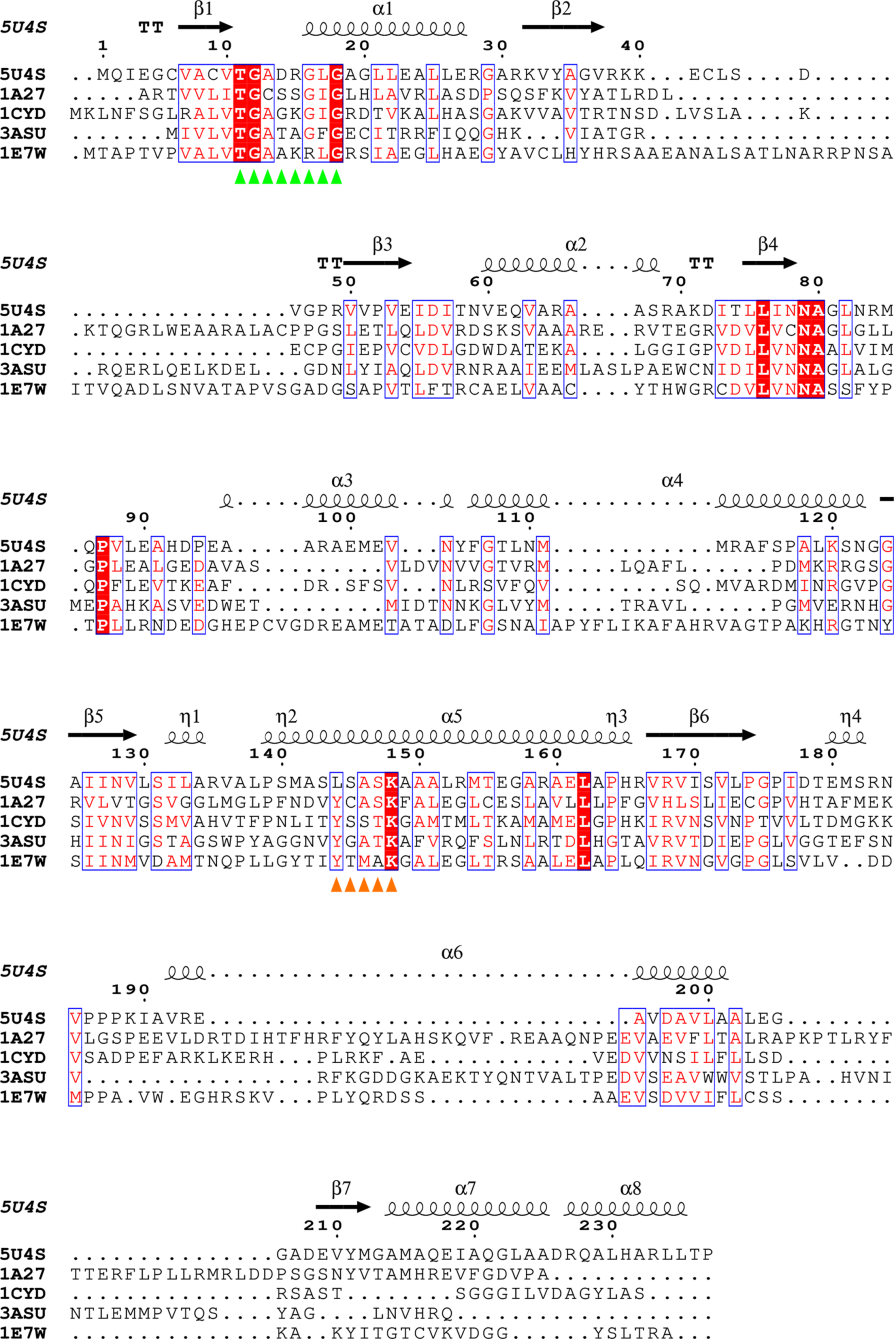
Sequence alignment of BcSDR with other NADP-binding SDRs. A sequence alignment of BcSDR (PDB entry 5u4s) with *Homo sapiens* 17-β-hydroxysteroid dehydrogenase (PDB entry 1a27, 30.15% sequence identity to PDB entry 5u4s; Mazza, 1997 ▸), *Mus musculus* carbonyl reductase (PDB entry 1cyd, 32.82% identity; Tanaka *et al.*, 1996 ▸), *Escherichia coli* serine dehydrogenase (PDB entry 3asu, 27.42% identity; Yamazawa *et al.*, 2011 ▸) and *Leishmania major* pteridine reductase (PDB entry 1e7w, 25.69% identity; Gourley *et al.*, 2001 ▸) is shown. The green triangles show the NADP-binding sequence motif and the orange triangles show the active-site sequence motif. This figure was prepared with *ESPript* 3.0 (Robert & Gouet, 2014 ▸).

SDRs are known to be NAD(P)-dependent enzymes. The structure of BcSDR showed clear electron density for NADP^+^ in the coenzyme-binding site in both chains (Fig. 3 ▸*a*). NADP^+^-binding SDRs are governed by the presence of a basic residue within the glycine-rich coenzyme-binding motif (Kavanagh *et al.*, 2008 ▸). They also can have a basic residue at the first position after β-strand 2 (Kallberg *et al.*, 2002 ▸, 2010 ▸). BcSDR has the conserved TG*xxx*G*x*G motif (Fig. 4 ▸) representative of the classical SDR subfamily. The Asp14 and Arg15 residues within this motif, as well as the nearby Arg38, make key hydrogen-bonding interactions with the phosphate of NADP (Fig. 3 ▸*b*). In this case, Arg15 is the basic residue within the glycine-rich motif, while Arg38 is the basic residue at the first position after β-strand 2. The NAD-dependent SDRs also tend to have a more enclosed coenzyme-binding region, with a nearby helix and loop structure occupying the space where the phosphate would bind, thereby imparting a sterically selective force for NAD binding (Fig. 3 ▸*c*).

**Figure 5:**
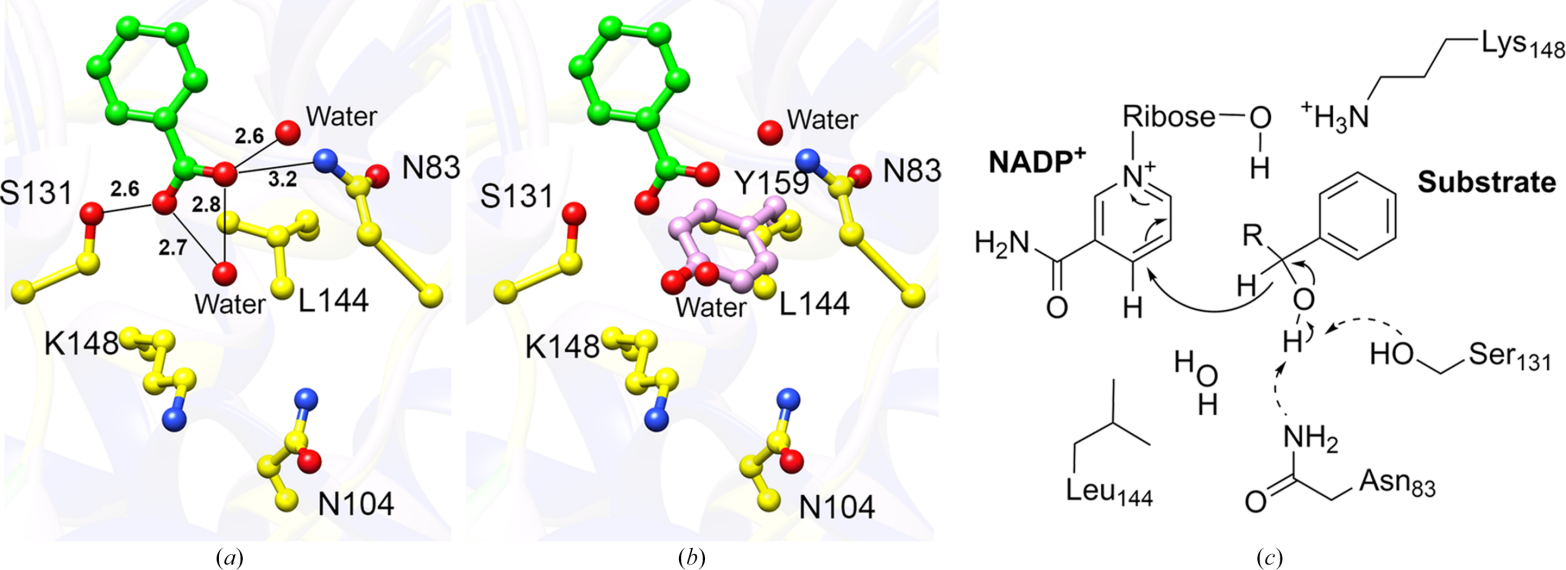
Active site of BcSDR. The active site of BcSDR (yellow C atoms) in complex with benzoic acid (green C atoms) is shown in (*a*). Water molecules are represented as red spheres. Hydrogen bonds are illustrated with black lines and bond distances are given in Å. O atoms are shown in red and N atoms are shown in blue. BcSDR and a *B. multivorans* SDR with the active-site Leu144 (yellow) and Tyr159 (pink) residues overlaid (*b*) reveal a similar environment between the water molecule and the O atom of Tyr159. Because BcSDR does not have the conserved tyrosine of classical SDRs, it is likely that Ser131 or Asn83 (*c*) would act as the general base in the enzyme mechanism.

**Figure 6:**
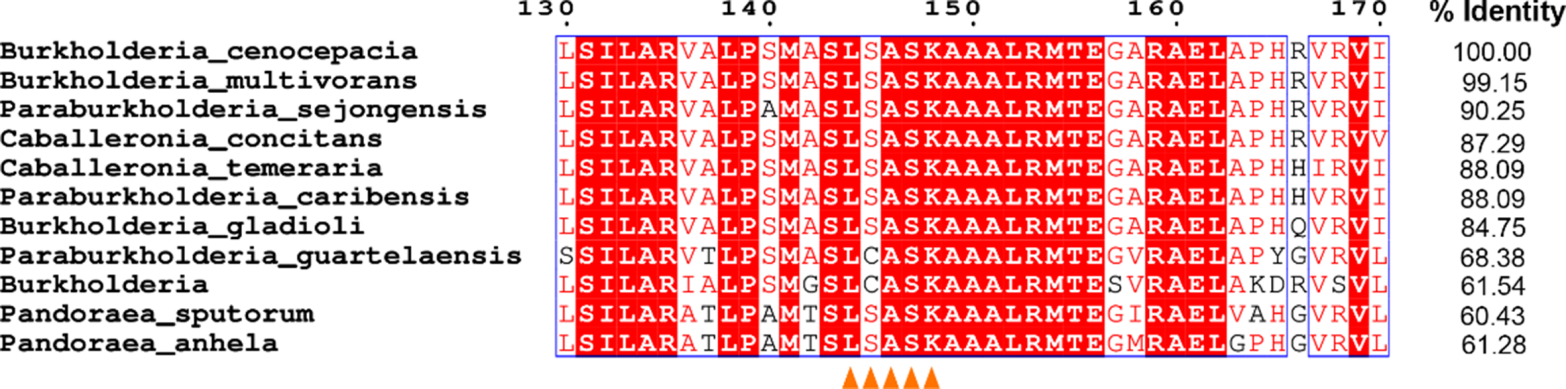
Alignment of several SDR sequences from the order Burkholderiales. An alignment of ten SDR sequences from different Burkholderiales bacteria with that of BcSDR shows a high degree of sequence conservation. The active site, highlighted with orange triangles, shows that the L*xxx*K motif observed in BcSDR is common among other members of Burkholderiales and is distinct from the canonical SDR motif.

In addition to the coenzyme, BcSDR co-crystallized with a molecule of benzoic acid bound in the active site (Figs. 5 ▸*a* and 5 ▸*b*). Benzoic acid was not added during crystallization, nor was it present in any of the media or purification buffers. This suggests that this molecule is likely to be representative of the native ligand structure. The benzoic acid in the BcSDR structure is stabilized by hydrogen bonds to Asn83, Ser131 and two water molecules (Fig. 5 ▸*a*). Catalysis in the substrate-binding domain typically involves a Y*xxx*K sequence motif in helix 5 and upstream asparagine and serine amino-acid residues. The tyrosine residue in this motif is highly, but not strictly, conserved and is the catalytic base in the majority of SDRs (Kavanagh *et al.*, 2008 ▸). BcSDR is one of the few exceptions to this rule, and instead has an L*xxx*K motif (Fig. 4 ▸). The tyrosine residue in this conserved motif is believed to be part of the catalytic tetrad Asn–Ser–Tyr–Lys characteristic of SDRs. The tyrosine residue putatively initiates the proton transfer to the substrate in the proposed mechanism (Filling *et al.*, 2002 ▸). In the structure of BcSDR, a leucine residue (Leu144) and a well ordered water molecule are present at the position that the conserved tyrosine would typically occupy (Fig. 5 ▸*b*). As the leucine residue would be unable to function similarly to the tyrosine, this observation suggests that BcSDR employs a different catalytic mechanism to that currently proposed for other SDRs. In this case, it is likely that either Ser131 or Asn83 would act as the base (Fig. 5 ▸*c*). Further studies are needed to fully elucidate the mechanism of BcSDR. To ensure that this sequence is not an artifact, or unique to this particular subspecies, we conducted a *BLAST* search (Altschul *et al.*, 1990 ▸), using the sequence of BcSDR, across the Burkholderiales class. Our results (Fig. 6 ▸) indicate that this L*xxx*K motif is conserved across a wide range of species, which indicates that this tyrosine-to-leucine substitution is not unique to this subspecies and could be a functionally relevant difference that distinguishes this class of organism. Despite BcSDR having an oligomeric structure and a coenzyme-binding motif that are suggestive of a classical SDR, the unusual active-site residues indicate that this protein is more appropriately classified as a divergent SDR (Kallberg *et al.*, 2010 ▸; Kavanagh *et al.*, 2008 ▸).

**Figure 7:**
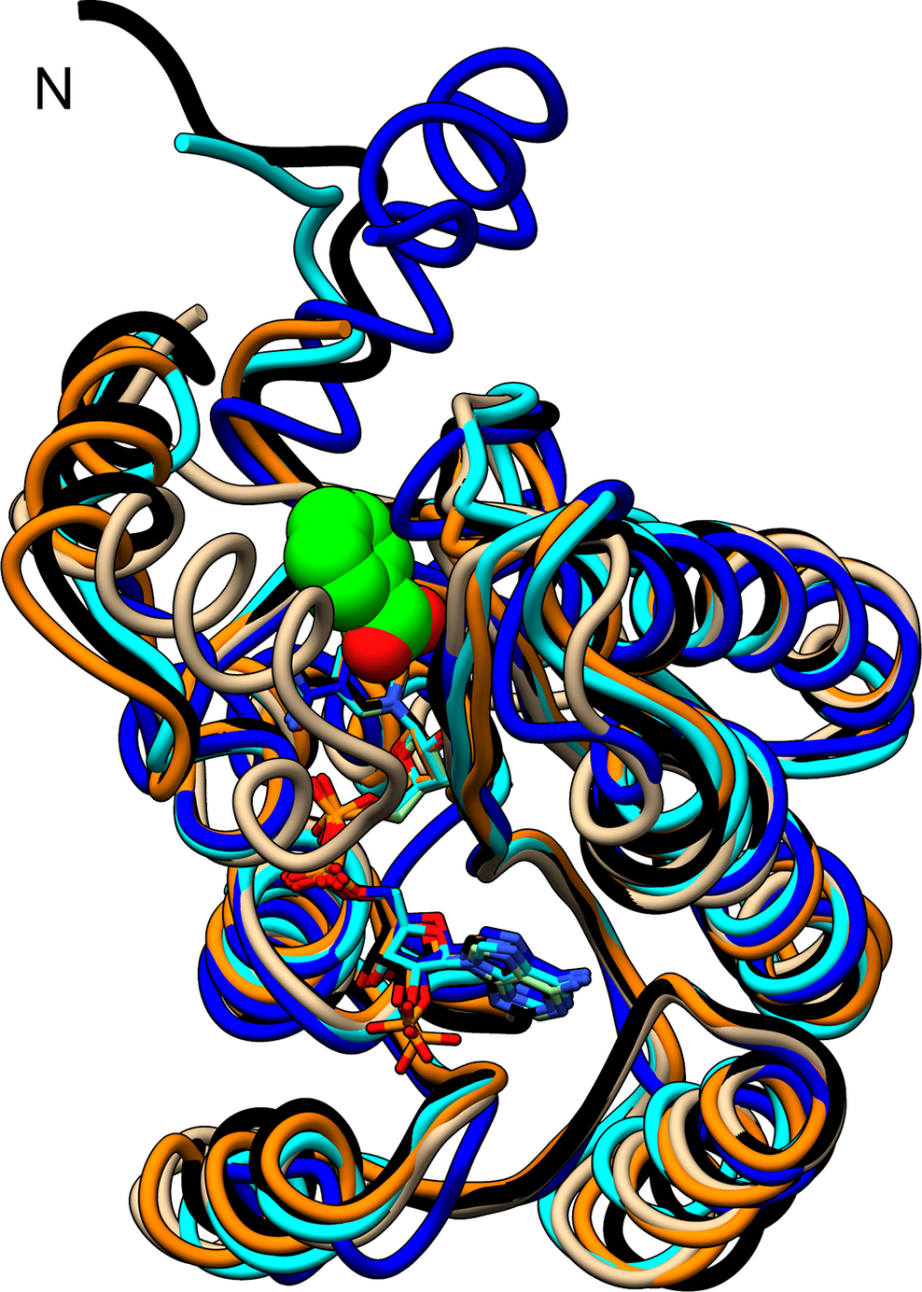
Superposition of BcSDR on its four closest structural homologs. The active-site cavity is defined by a space-filled representation of benzoic acid. The superimposed proteins (BcSDR in blue, PDB entry 6vsp from *Serratia marcescens* in black, PDB entry 1nff from *Mycobacterium tuberculosis* in cyan, PDB entry 2jah from *Streptomyces clavuligerus* in tan and PDB entry 4nbu from *Bacillus* sp. SG-1 in orange; Javidpour *et al.*, 2014 ▸; MacKenzie *et al.*, 2007 ▸; Subramanian *et al.*, 2020 ▸; Yang *et al.*, 2003 ▸) demonstrate the overall structural similarity and highlight the differences in the substrate-binding cavities necessary for these SDRs to catalyse their distinct chemistries.

A structural similarity search using the *DALI* server (Holm *et al.*, 2023 ▸) revealed proteins predominantly annotated as SDRs as the top hits with similarity to BcSDR (Table 5 ▸). An overlay of BcSDR and the top four proteins from the *DALI* search shows, as expected, a high degree of overall structural conservation, with some differences observed in the active-site cavity (Fig. 7 ▸). This structural variety in the active site is characteristic of the broad range of substrates acted upon by SDRs. The results of the structure-similarity search using BcSDR are consistent with the inherent substrate variability within this protein family. Amongst the top hits are dehydrogenases that bind linear alcohols (2,3-butanediol), sugar alcohols (galactitol), sulfonates (hydroxypropylethane thiosulfonate), β-lactams (clavulanic acid), steroids (estradiol) and prosthetic groups on proteins (acyl carrier protein).

## 4. Conclusion

The structure reported here expands our understanding of SDR enzymes and provides a valuable structural framework for future studies of the role that this enzyme plays in the life cycle of *B. cenocepacia*. Despite having the classical SDR fold and the conserved coenzyme-binding domain, the distinct active-site architecture of BcSDR suggest that this protein is most appropriately classed as a divergent SDR. Further studies will be needed to better understand how this enzyme can catalyse a dehydrogenase reaction in the absence of the highly conserved tyrosine residue.

## Supplementary Material

PDB reference: short-chain dehydrogenase from *Burkholderia cenocepacia* J2315, 5u4s

## Figures and Tables

**Table 1:** Macromolecule-production information

Source organism	*Burkholderia cenocepacia* (strain J2315)
DNA source	Jane L. Burns, Seattle Children’s Pediatrics
Forward primer	5′-CTCACCACCACCACCACCATATGGTGGCTTACGATCTTCAGGGCAAATTGAAGGCTGCGTGGC-3′
Reverse primer	5′-ATCCTATCTTACTCACTTAAGGCGTCAAAAGGCGCGCG-3′
Expression vector†	pBG1861
Expression host	*Escherichia coli* BL21(DE3) R3 Rosetta
Complete amino-acid sequence of the construct produced	MAHHHHHHMQIEGCVACVTGADRGLGAGLLEALLERGARKVYAGVRKKECLSDVGPRVVPVEIDITNVEQVARAASRAKDITLLINNAGLNRMQPVLEAHDPEAARAEMEVNYFGTLNMMRAFSPALKSNGGAIINVLSILARVALPSMASLSASKAAALRMTEGARAELAPHRVRVISVLPGPIDTEMSRNVPPPKIAVREAVDAVLAALEGGADEVYMGAMAQEIAQGLAADRQALHARLLTP

† The expression clone and purified protein are available at https://targetstatus.ssgcid.org/Target/BuceA.00010.y.

**Table 2:** Crystallization

Method	Vapor diffusion, sitting drop
Temperature (K)	290
Protein concentration (mg ml^−1^)	23.45
Buffer composition of protein solution	20 m*M* HEPES pH 7.0, 300 m*M* NaCl, 5% glycerol, 1 m*M* TCEP
Composition of reservoir solution	Rigaku Reagents JCSG+ screen C6: 40% PEG 300, 0.1 *M* sodium phosphate dibasic/citric acid pH 4.2
Volume and ratio of drop	0.4 µl protein + 0.4 µl reservoir (1:1)
Volume of reservoir (µl)	80

**Table 3:** Data collection and processing Values in parentheses are for the outer shell.

Diffraction source	21-ID-G, APS
Wavelength (Å)	0.97856
Temperature (K)	100
Detector	Rayonix MX-300 CCD
Space group	*P*2_1_2_1_2_1_
*a*, *b*, *c* (Å)	39.27, 75.66, 146.01
α, β, γ (°)	90, 90, 90
Resolution range (Å)	50.0–1.40 (1.44–1.40)
Total No. of reflections	494506
No. of unique reflections	86241
Completeness (%)	99.5 (94.7)
Multiplicity	5.73 (3.61)
〈*I*/σ(*I*)〉	13.91 (2.12)
*R*_r.i.m_ †	0.079 (0.637)
Overall *B* factor from Wilson plot (Å^2^)	12.61

† Estimated *R*_r.i.m._ = *R*_merge_ [*N*/(*N* − 1)]^1/2^, where *N* is the data multiplicity.

**Table 4:** Structure solution and refinement Values in parentheses are for the outer shell.

Resolution range (Å)	50.00–1.40 (1.44–1.40)
Completeness (%)	99.5
σ Cutoff	*F* > 1.350σ(*F*)
No. of reflections, working set	86226
No. of reflections, test set	1984
Final *R*_cryst_	0.148 (0.236)
Final *R*_free_	0.166 (0.255)
No. of non-H atoms	
Protein	3469
Ligand	119
Water	450
Total	4038
R.m.s. deviations	
Bond lengths (Å)	0.008
Angles (°)	1.076
Average *B* factor (Å^2^)	12.61
Ramachandran plot	
Most favored (%)	98
Allowed (%)	2

**Table 5:** Results of a *DALI* search using the structure of BcSDR (June 2024)

PDB code	*Z*-score†	R.m.s.d.‡ (Å)	% ID§	Description	Organism
6vsp	29.8	2.1	22	2,3-Butanediol dehydrogenase	*Serratia marcescens*
1nff	29.0	2.3	27	Putative oxidoreductase RV2002	*Mycobacterium tuberculosis*
2jah	29.0	1.8	29	Clavulanic acid dehydrogenase	*Streptomyces clavuligerus*
4nbu	28.9	1.9	26	3-Oxoacyl-(acyl-carrier-protein) reductase	*Bacillus* sp. SG-1
6d9y	28.8	1.9	23	Short-chain dehydrogenase	*Paraburkholderia phymatum* STM815
3p19	28.6	1.8	26	Putative blue fluorescent protein	*Vibrio vulnificus*
8y11	28.6	1.9	21	SDR-family reductase	*Herbaspirillum huttiense*
2cfc	28.6	1.8	27	2-(*R*)-Hydroxypropylethane thiosulfonate dehydrogenase	*Xanothobacter autotrophicus* Py2
3lqf	28.5	1.8	29	Galactitol dehydrogenase	*Cereibacter sphaeroides*
5ig2	28.4	2.4	23	Short-chain dehydrogenase	*Paraburkholderia phymatum* STM815
4cqm	27.9	1.8	22	Estradiol 17-β-dehydrogenase 8	*Homo sapiens*

† Calculated *Z*-score for alignment.

‡ Root-mean-square deviation.

§ Percentage sequence identity between BcSDR and the listed protein.
