# Crystal structure of a short-chain dehydrogenase from *Burkholderia cenocepacia* J2315 in complex with NADP^+^ and benzoic acid. Corrigendum

**DOI:** 10.1107/S2053230X25001682

**Published:** 2025-03-01

**Authors:** Kafi K. J. Belfon, Olive Beyer, Jan Abendroth, David M. Dranow, Donald D. Lorimer, Ariel Abramov, Yazmine Latimore, Connor Hamilton, Aniah Dawkins, Isabella Hinojosa, Xavier Martinez, Sofia de Lourdes Mirabal, Miriam Duncan, Reagan Womack, Lillian Hicks, Zachary R. Turlington, Thomas E. Edwards, Andrew T. Torelli, Katherine A. Hicks, Jarrod B. French

**Affiliations:** a The Hormel Institute University of Minnesota Austin MN55912 USA; b Department of Chemistry and Biochemistry University of Maryland Baltimore County Baltimore MD21250 USA; c UCB BioSciences, Bainbridge Island, WA98110, USA; d Seattle Structural Genomics Center for Infectious Disease (SSGCID), Seattle, WA98105, USA; e Department of Natural Sciences Albany State University Albany GA31707 USA; f College of Health Professions, Nursing and Pharmacy Manchester University North Manchester IN46962 USA; g Department of Chemistry and Biochemistry Hampton University Hampton VA23668 USA; h Department of Integrative Biology Oklahoma State University Stillwater OK74078 USA; i Department of Biological Sciences College of the Sequoias Visalia CA93277 USA; j Department of Microbiology, Immunology and Molecular Genetics University of California Los Angeles Los Angeles CA90095 USA; k Biological and Chemical Sciences Department, Manhattan University, Bronx, NY10471, USA; l College of Natural Sciences and Mathematics Lenoir-Rhyne University Hickory NC28601 USA; m Department of Chemistry Clemson University Clemson SC29634 USA; n State University of New York at Cortland Cortland NY13045 USA; o Department of Chemistry Ithaca College Ithaca NY14850 USA

**Keywords:** short-chain dehydrogenase/reductases, research experiences for undergraduates, SSGCID

## Abstract

The name of one of the authors in the article by Belfon *et al.*[(2024), *Acta Cryst.* F**80**, 348–355] is corrected.

## Abstract 2

The article by Belfon *et al.*[(2024), *Acta Cryst.* F**80**, 348–355] is corrected.

In the article by Belfon *et al.* (2024 ▸) the name of one of the authors was given incorrectly. The correct name is Sofia de Lourdes Mirabal as given here.
